# Glycyrrhizin attenuates renal inflammation in a mouse Con A-hepatitis model via the IL-25/M2 axis

**DOI:** 10.1080/0886022X.2024.2356023

**Published:** 2024-05-24

**Authors:** Lingyun Li, Yuanyue Zhang, Zhongyan Wang, Xiangyu Chen, Min Fang

**Affiliations:** a Department of Gastroenterology, Affiliated Hangzhou First People’s Hospital, Westlake University School of Medicine, Hangzhou, China; b Department of Immunology, School of Basic Medicine, Tongji Medical College, Huazhong University of Science and Technology, Wuhan, China; c Department of Laboratory Medicine, Weifang Medical University, Weifang, China

**Keywords:** Glycyrrhizin, renal inflammation, Con A, IL-25, type 2 macrophages

## Abstract

Glycyrrhizin (GL) has immunoregulatory effects on various inflammatory diseases including hepatitis and nephritis. However, the mechanisms underlying the anti-inflammatory effect of GL on renal inflammation are not fully understood. Hepatorenal syndrome (HRS) is a functional acute renal impairment that occurs in severe liver disease, and we found that kidney injury also occurs in Con A-induced experimental hepatitis in mice. We previously found that GL can alleviate Con A-induced hepatitis by regulating the expression of IL-25 in the liver. We wanted to investigate whether GL can alleviate Con A-induced nephritis by regulating IL-25. IL-25 regulates inflammation by modulating type 2 immune responses, but the mechanism by which IL-25 affects kidney disease remains unclear. In this study, we found that the administration of GL enhanced the expression of IL-25 in renal tissues; the latter promoted the generation of type 2 macrophages (M2), which inhibited inflammation in the kidney caused by Con A challenge. IL-25 promoted the secretion of the inhibitory cytokine IL-10 by macrophages but inhibited the expression of the inflammatory cytokine IL-1β by macrophages. Moreover, IL-25 downregulated the Con A-mediated expression of Toll-like receptor (TLR) 4 on macrophages. By comparing the roles of TLR2 and TLR4, we found that TLR4 is required for the immunoregulatory effect of IL-25 on macrophages. Our data revealed that GL has anti-inflammatory effects on Con A-induced kidney injury and that the GL/IL-25/M2 axis participates in the anti-inflammatory process. This study suggested that GL is a potential therapeutic for protecting against acute kidney injury.

## 1. Introduction

Hepatorenal syndrome (HRS) is a form of kidney function impairment that is characteristic of patients with cirrhosis [1]. In the clinic, acute kidney injury (AKI) is observed in acute-on-chronic liver failure (ACLF) patients [2]. This interesting phenomenon also occurs in a Con A-induced mouse model of experimental hepatitis. Con A is known to mitogenically activate T lymphocytes and is used to induce liver injury in mice. Although Con A-induced liver injury has been well studied, there have been few studies on the effects of Con A on the kidney. In our previous studies on Con A-induced hepatitis, we observed that Con A could also cause renal inflammation. Similarly, it has been observed that the administration of Con A induces an increase in the expression of IFN-γ and TNF-α in the kidney [3].

Glycyrrhizin (GL) is purified from licorice and is clinically used to treat inflammation and viral diseases [4]. In particular, GL has been widely used to treat chronic hepatitis B in Japan [5,6]. In our previous research on Con A-induced hepatitis, GL dramatically alleviated inflammation in the liver [22]. However, compared with the large number of studies on GL in hepatitis, the mechanisms underlying the anti-inflammatory effect of GL on the kidney are still not fully understood. In an experimental mouse model, it has been shown that the overexpression of HMGB1, Toll-like receptor (TLR) 4, NF-κB and the glomerular injury marker nestin in the setting of diabetic kidney disease was significantly ameliorated by GL administration [7]. Other research has shown that GL has a protective effect against renal injury and dysfunction by inhibiting HMGB1 and reducing oxidative stress [8]. Given the close relationship between liver and kidney function, we sought to further clarify whether GL also has an effect on Con A-induced nephritis.

Macrophage infiltration is a notable pathological change in the kidneys of Con A-treated mice [9]. Paeoniflorin exerts a protective effect on Con A-induced renal damage in mice by inhibiting macrophage infiltration [9]. Macrophages play an important role in tissue homeostasis and immune responses in normal and diseased kidneys [10]. In mice, Con A administration induces macrophage infiltration, causing tissue damage in both the liver and kidney [8]. Two distinct macrophage phenotypes (proinflammatory M1 and anti-inflammatory M2 macrophages) contribute to kidney injury and repair during the progression of renal interstitial fibrosis [11]. M2 macrophage-based therapy is a promising approach for chronic kidney disease [12]. Multiple cytokines and signaling pathways, including TLR4 signaling, are involved in the polarization of macrophages [13,14].

Interleukin-25 (IL-25) (also called IL-17E), another IL-17 family cytokine, is well known to regulate allergic responses and type 2 immunity [15]. IL-25 is highly expressed by polarized T helper (Th) 2 cells and plays a key role in the expansion of Th2 cell responses in various organs [16]. On the other hand, IL-25 can target and deliver negative signals to macrophages and T helper cells by suppressing the production of proinflammatory cytokines [17,18]. According to several previous reports, IL-25 protects against renal injury in individuals with drug-induced kidney disease by inducing M2 macrophages or eliciting group 2 innate lymphoid cells and multipotent progenitor type 2 cells [19–21].

In our previous study, we reported that GL alleviates Con A induced hepatitis by reducing the production of IL-17 and enhancing the expression of IL-25 [22]. However, whether GL can alleviate Con A-induced nephritis remains unclear. We hypothesize that GL may promote the differentiation of M2 macrophages and alleviate Con A-induced nephritis by regulating the expression of IL-25 in the kidneys. Therefore, in this study, we aimed to explore whether GL affects the progression of Con A-induced nephritis by regulating the IL-25/M2 axis.

## 2. Materials and methods

### 2.1. Animals

Male C57BL/6J mice were purchased from Beijing HFK Bioscience (China). C57BL/6.129-Tlr2^tmIkir/JNju^ (TLR2^–/–^) mice and C57BL/10^ScN/JNju^ (TLR4^–/–^) mice were purchased from the Biomedical Research Institute (Nanjing, China), and only male mice were used in the studies. All the mice used were 7–8 weeks old (22–24 g). Mice were maintained under specific pathogen-free (SPF) conditions and studied in compliance with the animal care and use committee guidelines of Tongji Medical College, HUST (China). The study protocols were specifically reviewed and approved by this ethics committee.

### 2.2. Major materials

Con A with undetectable endotoxin was purchased from Sigma-Aldrich (Saint Louis, MO, USA) and injected into mice via the tail vein at a dose of 15 mg/kg. GL was purchased from Tokyo Chemical Industry (Tokyo, Japan) and administered to mice by intraperitoneal injection at a dose of 50 mg/kg 1 h before Con A injection. Recombinant mouse IL-25 was purchased from Sino Biological Inc (Wuhan, China). IL-25 was intraperitoneal injected at a dose of 100 μg per mouse 1 h before Con A injection in the *in vivo* experiment and at a concentration of 50 μg/ml in 1640 medium (Gibco) supplemented with 10% FBS (Gibco) in the *in vitro* experiment. An anti-IL-25 antibody was purchased from Abcam (Shanghai, China). The urea and blood creatinine testing kits were purchased from Nanjing Jiancheng (Nanjing, China). APC-conjugated anti-mouse TLR4 antibody and PE-conjugated anti-mouse F4/80 antibody were purchased from Biolegend (San Diego, CA, USA), and the anti-mouse CD206 antibody was purchased from Sevicebio (Wuhan, China). ELISA kit for IL-1β and IL-10 were purchased from Biolegend.

### 2.3. Con A-induced tissue damage in mice

Mice were pretreated with IL-25 and received Con A injection 1 h later. After 10 h, urine and blood were harvested. Mice were euthanized under 2% pentobarbital sodium, and all efforts were made to minimize suffering; kidney and liver samples were taken for the following investigations.

### 2.4. Histology analysis

Briefly, kidney and liver specimens from each group were preserved in 4% paraformaldehyde and dehydrated in a graded ethanol series. The samples were cut into 4 μm-thick sections and then subjected to hematoxylin–eosin (HE) staining or periodic acid Schiff staining (PAS) staining. The surface area of the necrotic regions in liver was measured by Image-Pro Plus 6.0., and the necrotic regions were counted according to a previous method [23]. Six random fields were selected and assessed for pathological kidney damage using a double-blind method based on the following criteria. Based on the type of kidney injury in this study, we have established a standard for pathological grading of the kidney injury: 1 = No alteration in glomerular morphology, absence of hemorrhage, no tubular edema, and no infiltration of inflammatory cells; 2 = Mild enlargement of glomeruli, mild hemorrhage, mild tubular edema, and mild infiltration of inflammatory cells; 3 = Marked enlargement of glomeruli, severe hemorrhage, marked tubular edema, and marked infiltration of inflammatory cells; 4 = Disorganization of glomerular and tubular structures with necrosis of tubular epithelial cells and extensive infiltration of inflammatory cells.

### 2.5. Protein molecule and cytokine evaluation

The serum levels of ALT were tested by an automatic biochemical analyzer, and the levels of creatinine (Cr) in the urine and serum were measured by a testing kit. IL-1β and IL-10 in the culture medium were evaluated by ELISA.

### 2.6. Cell culture

Peritoneal macrophages were cultured in 10% FBS 1640 medium for 8–10 h, after which the nonadherent cells were removed. The macrophages were sequentially challenged with Con A or IL-25 for 10 h.

### 2.7. Immunohistochemistry (IHC)

Briefly, the embedded tissue was deparaffinized in xylene and rehydrated. Then, 3% H_2_O_2_ in PBS was used to quench the peroxidase for 15 min at room temperature. 5% BSA was used to block the sections for 30 min at 37 °C. Then, the sections were incubated with the anti-IL-25 antibody overnight at 4 °C followed by species-specific biotinylated secondary antibodies at room temperature for 30 min. After washing, the sections were stained with DAB. Counterstaining was performed with hematoxylin before dehydration and mounting.

### 2.8. Immunofluorescence (IF)

Single staining for CD206 was performed on paraffin sections. The slices were deparaffinized in xylene, rehydrated and incubated with protease K (20 μg/ml) for 10 min at 37 °C. 5% BSA was used to block the sections for 30 min at 37 °C. Then, the sections were incubated with the anti-CD206 antibody overnight at 4 °C and followed by the secondary PE-conjugated AffiniPure goat anti-rabbit antibody for 30 min at room temperature in the dark. Then, the nuclei were stained with DAPI.

### 2.9. Flow cytometry

Cultured macrophages were scraped from dishes. Subsequently, the cells were stained with surface markers (F4/80 and TLR4) for 30 min at 4 °C, followed by flow cytometry analysis. Flow cytometry data were collected with a BD LSRFortessa cytometer and analyzed with FlowJo X.

### 2.10. Quantitative real-time PCR

Total RNA samples from kidney tissues were isolated using the TRIzol® Plus RNA Purification Kit (Thermo Fisher Scientific, Waltham, MA, USA). cDNA was synthesized from 1 µg RNA using SuperScript™III First-Strand Synthesis SuperMix (Thermo Fisher Scientific). The mRNA levels of IL-6, IL-17A, CD206 were detected by real-time PCR using Power SYBR® Green PCR Master Mix (Applied Biosystems, MA, USA). GAPDH was used as the standardized control. Relative mRNA levels were calculated using the 2^–△△CT^ method. The primers used are as follows:

GAPDH, 5′-GAAGGTCGGTGTGAACGGATTTG-3′, 5′-CATGTAGACCATGTAGTTGAGGTCA-3′; IL-6, 5′-GAGACTTCCATCCAGTTGCC-3′, 5′-AAGTGCATCATCGTTGTTCATACA-3′; IL-17A, 5′-CACCGCAATGAAGACCCTGATA-3′, 5′-CCAGGATCTCTTGCTGGATGAGA-3′; CD206, 5′-CAGCAAGTGATTTGGAGGCTGA-3′, 5′-CCGTAAGCCCAATTTTCATAGGA-3′.

### 2.11. Statistical analysis

The experimental data are presented as the means ± SD. Student’s *t* test was used to analyze the differences between groups. Two-sided probability (*p*) values <0.05 were considered significant.

## 3. Results

### 3.1. The administration of Con A induces both hepatitis and kidney injury in mice

**Figure 1.**
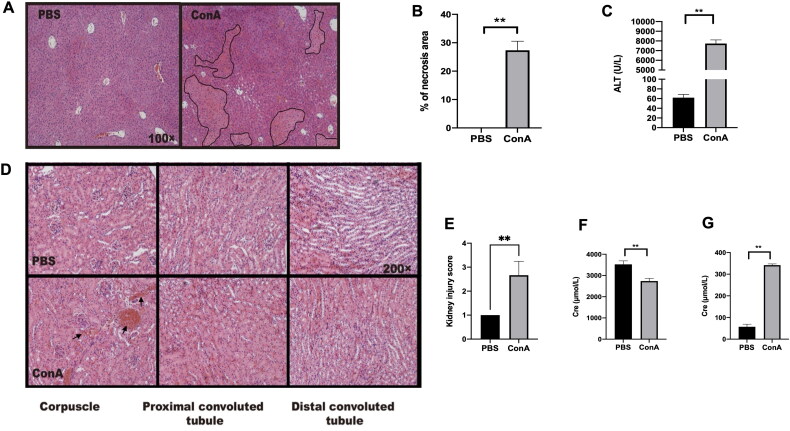
Renal inflammation in mice with Con A-induced hepatitis. C57BL/6 mice were challenged (via tail vein injection) with Con A at 15 mg/kg body weight. Ten hours after injection, the serum was collected to measure alanine aminotransferase (ALT) and creatinine levels. The livers and kidneys were excised for hematoxylin/eosin (HE) staining analysis. (A) Graph showing liver injury after Con A challenge (HE staining, original magnification, ×100). (B) Statistical analysis of necrotic areas in the liver. (C) Graph showing the level of ALT. (D) Kidney histology after Con A administration. (E) Kidney injury scores were evaluated based on HE staining. (F) The creatinine levels in the urine were measured after Con A injection. (G) The creatinine levels in the serum were measured after Con A injection. The data from one representative of three experiments are shown. Each group contained 3–5 mice, * *p* < 0.05, **p < 0.01.

We first assessed the effects of Con A challenge on the liver and kidney, and male C57BL/6 mice were administered Con A via i.v. injection. Compared with PBS control mice, Con A-challenged mice exhibited both hepatitis (Figure 1A–C) and kidney inflammation (Figure 1D–G). In the kidney, the kidney corpuscle and proximal convoluted tubule exhibited hemorrhage and swelling after Con A challenge (Figure 1D and E) as well as an increase in serum creatinine compared with those of control mice treated with PBS (Figure 1G). These results indicated that the administration of Con A leads to inflammation in the liver and kidney.

### 3.2. The application of glycyrrhizin attenuates Con A-induced inflammation in the kidney and enhances the expression of IL-25

**Figure 2.**
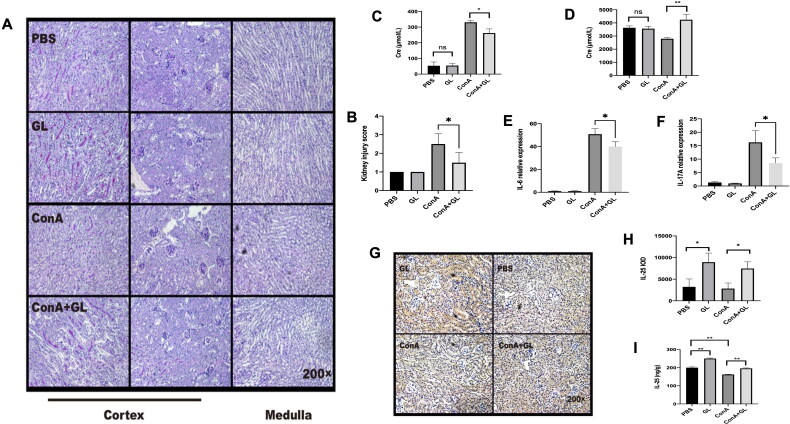
GL enhances the expression of IL-25 to protect against renal inflammation caused by Con A challenge. C57BL/6 mice were injected with Con A via the tail vein at a dose of 15 mg/kg body weight. GL was intraperitoneally injected 1 h before Con A injection. Kidney samples were harvested for immunohistochemistry (IHC) and PAS staining analysis 10 h after Con A injection. (A) PAS staining showing renal injury in different segments. (B) Kidney injury scores were evaluated based on PAS staining. (C) Serum creatinine levels were measured. (D) Creatinine levels in urine were measured. (E) Real-time PCR analysis of IL-6 in the kidney. (F) Real-time PCR analysis of IL-17A in the kidney. (G) IHC showing the expression of IL-25 in kidney samples from each group. (H) Image-Pro Plus 6.0 was used to analyze the integrated optical density (IOD) of IL-25 in kidney. (I) Evaluation of IL-25 in kidney tissue homogenates by ELISA. The values are the means ± SDs; *p < 0.05, **p < 0.01. All the data shown here are representative of independent experiments with similar results. The results of at least three independent experiments with 3–5 mice per group are shown.

Next, we examined the anti-inflammatory effect of GL on Con A-induced inflammation of the kidney. Pathology of the kidneys revealed that GL alleviated the injury of the kidney caused by Con A challenge (Figure 2A) and (B). In line with the pathological analysis, compared with Con A treatment, the application of GL to mice treated with Con A decreased the serum creatinine concentration (Figure 2C). At the same time, the level of creatinine in the urine was elevated upon GL administration (Figure 2D). IL-6 and IL-17 are typical pro-inflammatory cytokines. The results showed that Con A can cause a marked increase in the expression of IL-6 and IL-17 in the kidney, while GL can reduce the expression of IL-6 and IL-17 (Figure 2E and F). In our previous study, we found that GL can alleviate hepatitis by reducing IL-17 and promoting the expression of IL-25, which is known for promoting the type 2 immune responses [22]. Compared with PBS, GL enhanced the expression of IL-25 in the kidney and reversed the decrease in IL-25 caused by Con A challenge (Figure 2I). Consistently, immunohistochemistry analysis further confirmed the above results (Figure 2G and H). These results indicated that GL has a protective effect on Con A-induced inflammation in the kidney and that GL enhances the production of IL-25 in the kidney.

### 3.3. Treatment with IL-25 ameliorates Con A-induced inflammation of the kidney

**Figure 3.**
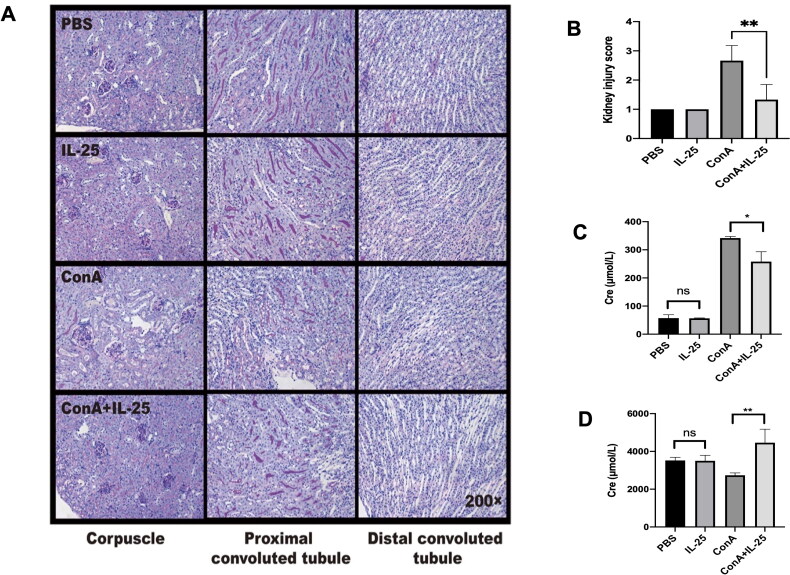
Administration of IL-25 protects against Con A-induced inflammation and functional impairment of the kidney in mice. C57BL/6 mice were injected with Con A via the tail vein at a dose of 15 mg/kg body weight. IL-25 was intraperitoneally injected 1 h before Con A injection. Kidney samples were harvested for PAS staining analysis 10 h after Con A injection. (A) Mice were administered Con A and/or IL-25, and kidney sections were stained with PAS. (B) Kidney injury scores were evaluated based on PAS staining. (C) The levels of creatinine in the serum were determined. (D) The levels of creatinine in urine were determined. The graphs show that IL-25 could ameliorate renal injury and functional impairment induced by Con A challenge. The values are the means ± SDs; *p < 0.05, **p < 0.01. The experiments were independently carried out at least 3 times, *n* = 3–5.

We next tested whether IL-25 has an inhibitory effect on inflammation in the kidney caused by Con A. As shown in Figure 3A and B, treatment with Con A plus IL-25 had a protective effect on kidney inflammation compared to Con A treatment alone. As supportive evidence, treatment with IL-25 decreased the serum creatinine level in mice challenged with Con A compared with that in mice challenged with Con A alone (Figure 3C) but significantly increased the creatinine concentration in the urine of animals injected with Con A alone (Figure 3D). These data showed that IL-25 has an anti-inflammatory effect on Con A-induced inflammation in the kidney.

### 3.4. IL-25 exerts anti-inflammatory effects by shaping the profile of macrophages

**Figure 4.**
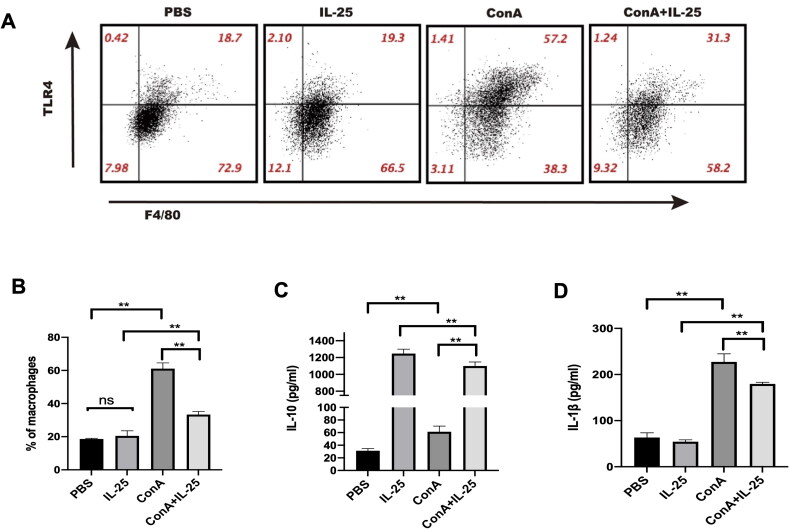
IL-25 inhibits Con A-induced expression of TLR4 and IL-1β and upregulates the production of IL-10 by macrophages. (A) TLR4 expression in peritoneal macrophages cultured with IL-25 and/or Con A for 10 h was detected by FACS. (B) The TLR4 receptor on macrophages was upregulated by Con A and downregulated by IL-25. (C) The levels of the cytokines IL-1β and IL-10 (D) in the culture medium were assessed, and IL-10 was significantly increased after IL-25 treatment. The values are the means ± SDs; *p < 0.05, **p < 0.01. The experiments were independently carried out at least 3 times, *n* = 3–5.

We next explored the possible molecular mechanisms by which IL-25 exerts anti-inflammatory effects on Con A-induced inflammation in the kidney. For this purpose, peritoneal macrophages were cultured in the presence of Con A alone, Con A combined with IL-25, IL-25 alone, or the PBS control. IL-25 downregulated the Con A-induced increase in TLR4 expression in macrophages (Figure 4A and B). In parallel, IL-25 augmented the secretion of the inhibitory cytokine IL-10 by macrophages but inhibited the expression of the inflammatory cytokine IL-1β by macrophages (Figure 4C and D). These results indicated that IL-25 exerts anti-inflammatory effects, at least in part, through regulating the profile of macrophages.

### 3.5. IL-25 promotes the polarization of type 2 macrophages

**Figure 5.**
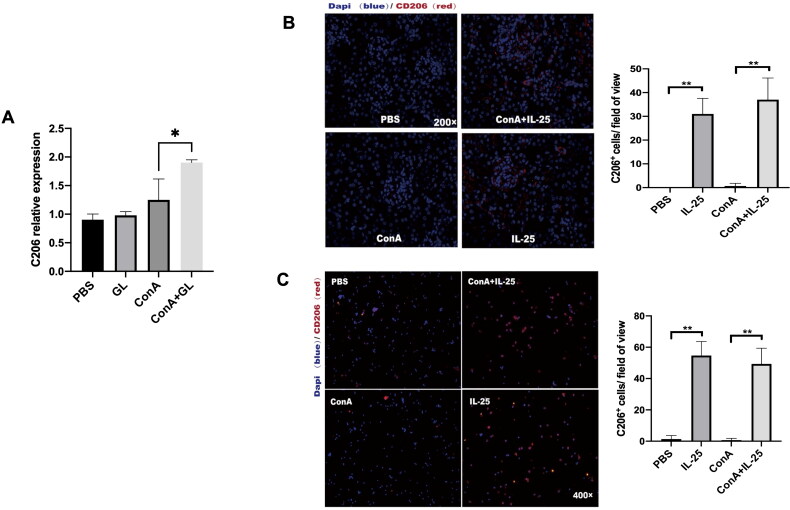
GL and IL-25 enhances the expression of CD206 in the kidney and in macrophages. (A) Real-time PCR analysis of CD206 in the kidney. CD206 expression was upregulated in the GL treatment groups. (B) IF staining of kidney sections from the different treatment groups following CD206 staining (red). CD206 expression was upregulated in the IL-25 treatment groups. (C) Peritoneal macrophages were cultured with IL-25 and/or Con A, and the CD206 receptor was detected by IF (red). The values are the means ± SDs; *p < 0.05, **p < 0.01. A representative experiment of at least three independent experiments with 3 mice per group is shown.

As type 2 macrophages (M2) have been demonstrated to have anti-inflammatory effects on the kidney [24], we further examined the phenotype of macrophages in mice treated with Con A combined with IL-25 or Con A alone. We used CD206 as a marker of M2 macrophages, and immunofluorescence staining revealed that, compared with Con A challenge alone, treatment with IL-25 dramatically enhanced the expression of CD206 in kidney sections (Figure 5B). The results of the investigation of peritoneal macrophages were consistent with those of the *in vivo* experiments (Figure 5C). These results suggested that IL-25 promotes the polarization of type 2 macrophages. To confirm whether GL could also promote the polarization of M2 macrophages, we detected the expression of CD206 in kidney. The results showed that, consistent with the trend of the action of IL-25, GL could also promote the production of M2 in Con A-induced nephritis (Figure 5A).

### 3.6. IL-25 enhances the polarization of type 2 macrophages by downregulating TLR4

**Figure 6.**
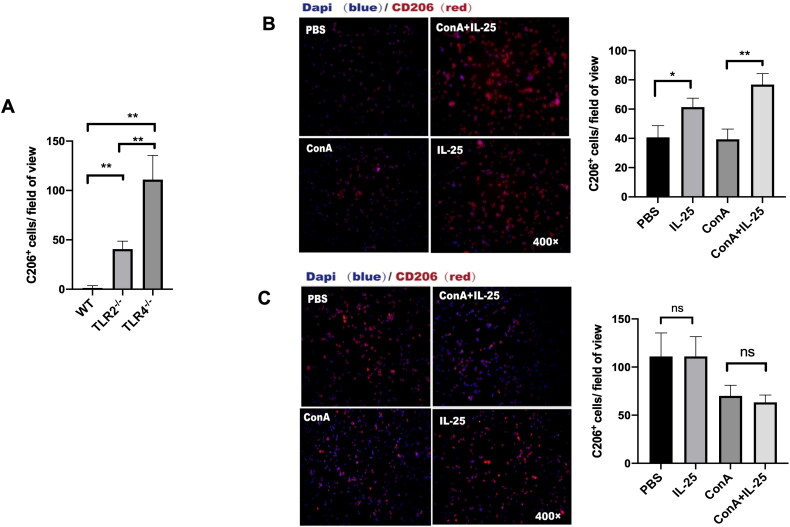
TLR4 is required for the IL-25-induced increase in CD206 expression by macrophages. (A) TLR4-deficient mice in the PBS group had more M2 macrophages than wild-type (Figure 5B) and TLR2-deficient mice (Figure 6B and C). (B) Peritoneal macrophages from TLR2^–/–^ mice were cultured *in vitro* and challenged with IL-25 or Con A. (C) Peritoneal macrophages from TLR4^–/–^ mice were cultured *in vitro* and challenged with IL-25 or Con A. After 10 h, the CD206 receptor (red) was detected by IF. The values are the means ± SDs; *p < 0.05, **p < 0.01. A representative experiment of at least three independent experiments with 3 mice per group is shown.

Finally, we investigated the role of TLR4 in the polarization of type 2 macrophages. A comparison of the PBS-treated TLR2^–/–^ mice and TLR4^–/–^ mice revealed that TLR4 deficiency led to a significant increase in the expression of CD206. We found that TLR4-deficient mice in the PBS group had more M2 macrophages than did wild-type and TLR2-deficient mice (Figures 6A). This result suggested that TLR4 signaling regulated the production of M2 macrophages. Furthermore, in TLR4-deficient mice, IL-25 had no influence on the polarization of M2 macrophages (Figure 6C). However, TLR2 deficiency did not affect the impact of IL-25 on M2 macrophages (Figure 6B). This result suggested that the IL-25-induced upregulation of M2 macrophages occurs in a TLR4-dependent manner. IL-25 promotes the polarization of M2 macrophages by downregulating TLR4.

**Figure 7.**
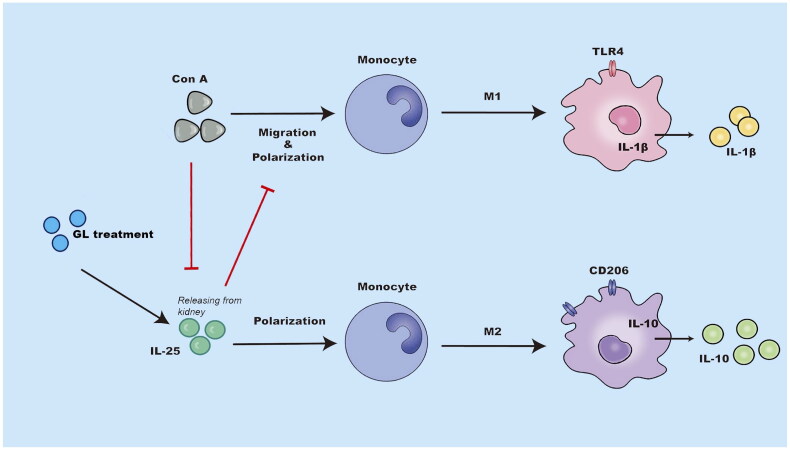
Schematic representation of the immunoregulatory role of IL-25 in Con A-induced inflammation of the kidney. The administration of glycyrrhizin enhances the production of IL-25, which is an anti-inflammatory cytokine, in the context of Con A challenge. IL-25 promotes the polarization of type 2 macrophages while inhibiting the generation of type 1 macrophages to restrain inflammation in the kidney elicited by Con A challenge..

## 4. Discussion

In the present study, we examined the effects and mechanisms of GL on kidney inflammation induced by Con A challenge in mice. We demonstrated that treatment with GL attenuated Con A-induced inflammation in the kidney. This anti-inflammatory effect was associated with enhancing the production of IL-25 by renal tissues, and IL-25 promoted the polarization of type 2 macrophages (Figure 7).

GL exhibits anti-inflammatory effects including protection against I/R-induced renal injury through the inhibition of inflammatory cell infiltration and the production of proinflammatory cytokines [25]. This protection is associated with the inhibition of the release of the endogenous inflammatory factor HMGB1 [26]. More recently, it was reported that the GL improves renal injury and inflammatory responses in diabetic rats by via regulating RAGE/TLR4-related ERK and p38 MAPK/NF-κB activation [27]. In line with the above observations, we found that GL protects against Con A-induced inflammation in the kidney. Importantly, a novel finding of this study is that GL enhances the expression of IL-25 in renal tissues.

IL-25 is an important immune regulator that can promote Th2 immune response-dependent immunity, inflammation, and tissue repair. Most attention has been given to the roles of IL-25 in allergic respiratory diseases, such as asthma and viral respiratory diseases, and IL-25 plays an important role in the beginning and progression of allergic diseases [15]. In recent years, the protective role of IL-25 in multiple diseases such as hepatic steatosis [28], atherosclerosis [29] and kidney injury [20,21], has been determined. IL-25 treatment reduced renal ischemic/reperfusion injury by eliciting type 2 innate lymphoid cells (ILC2s) and multipotent progenitor type 2 (MPP) (type2) cells in the kidney [20]. Furthermore, tubular-macrophage cross-talk occurs during kidney injury [30]. We showed that IL-25 reduces the Con A-induced renal injury by augmenting the generation of type 2 macrophages in the kidney.

The adult kidney has tissue-resident macrophages that can cause, prevent, and/or repair renal damage [31]. M1/M2 macrophage subtypes have been increasingly linked to inflammation in kidney and renal repair [32]. In the process of kidney injury, M1 macrophages promote inflammation and tubular cell apoptosis by secreting pathogenic factors such as IL-1β, TNF-α and IL-6; however, M2 macrophages reduce inflammation and promote renal recovery by secreting trophic factors such as IL-10, TGF-β and arginase-1 [33]. In the present study, we found that IL-25 promoted the generation of M2 cells in the kidney. IL-25 promoted the secretion of the inhibitory cytokine IL-10 and inhibited the expression of the inflammatory cytokine IL-1β by macrophages. It has been reported that IL-25 promotes hepatic macrophage differentiation into M2a macrophages both *in vivo* and *in vitro* via the IL-13/STAT6 pathway [28]. In the kidney, IL-25 also induces alternatively activated M2 macrophages, and IL-25 protects against renal injury in adriamycin nephropathy in mice by, at least in part, inducing Th2 immune responses [19].

In the present study, we also demonstrated that the expression of TLR4 in macrophages was increased in the Con A group and that IL-25 could downregulate the increase in TLR4 expression induced by Con A. TLR4 is a specific phenotype marker of M1 macrophages [34], and the activation of TLR4/NF-κB signaling can switch M2-polarized macrophages to M1-polarized macrophages in tumors [35,36]. In our study, TLR4- and TLR2-deficient mice were used to investigate the role of TLR4 in the polarization of M2 macrophages. We found that TLR4 (not TLR2) is important for sustaining the M1/M2 macrophage balance in the kidney. Moreover, IL-25 promoted the polarization of M2 macrophages by downregulating TLR4. Further studies are needed to investigate the molecular mechanism of macrophage polarization.

## 5. Conclusions

In conclusion, our findings demonstrated that GL can attenuate Con A-induced kidney injury by enhancing the production of IL-25 in renal tissues. IL-25 promotes the polarization of type 2 macrophages by downregulating the expression of TLR4 on macrophages. This study suggests that GL is a potential therapeutic for protecting against acute kidney injury.

## Data Availability

The datasets used and/or analyzed during the current study are available from the corresponding author upon reasonable request.

## Abbreviations

GL: glycyrrhizin
TLR: toll-like receptor
IL: interleukin
HMGB1: high mobility group box 1
M2: type 2 macrophages
Con A: concanavalin A
